# Case Fatality Rate Related to Microcephaly Congenital Zika Syndrome and Associated Factors: A Nationwide Retrospective Study in Brazil †

**DOI:** 10.3390/v12111228

**Published:** 2020-10-29

**Authors:** Maria Conceição N. Costa, Luciana Lobato Cardim, Maria Gloria Teixeira, Mauricio L. Barreto, Rita de Cassia Oliveira de Carvalho-Sauer, Florisneide R. Barreto, Martha Suely Itaparica Carvalho, Wanderson K. Oliveira, Giovanny V. A. França, Eduardo Hage Carmo, Roberto F. S. Andrade, Moreno S. Rodrigues, Rafael V. Veiga, Juliane F. Oliveira, Qeren H. R. F. Fernandes, Larissa C. Costa, Giovanini E. Coelho, Enny S. Paixao

**Affiliations:** 1 Oswaldo Cruz Foundation, Center of Data and Knowledge Integration for Health (CIDACS), Gonçalo Moniz Institute, Salvador, Bahia ZC 41745-715, Brazil; mariacncosta@ufba.br (M.C.N.C.); lucianacardim@yahoo.com.br (L.L.C.); magloria@ufba.br (M.G.T.); mauricio@ufba.br (M.L.B.); florisneide@gmail.com (F.R.B.); itamartha@gmail.com (M.S.I.C.); wkoliveira@gmail.com (W.K.O.); ehcarmo@gmail.com (E.H.C.); randrade@ufba.br (R.F.S.A.); rodriguesmsb@gmail.com (M.S.R.); rafaelvalenteveiga@gmail.com (R.V.V.); julianlanzin@gmail.com (J.F.O.); larissacatharina@gmail.com (L.C.C.); 2 Collective Health Institute, Federal University of Bahia, Salvador, Bahia ZC 40110-040, Brazil; ritacarvalhosauer@gmail.com; 3 East Regional Health Center of the State Health Secretariat of Bahia, Santo Antonio de Jesus, Bahia ZC 44570-550, Brazil; 4 Municipal Health Secretariat of Salvador, Bahia ZC 40010-010, Brazil; 5 Technical Directorate of Education and Research, Ministry of Defense Hospital das Armed Forces, Brasília ZC 70675-731, Brazil; 6 Secretariat of Health Surveillance, Ministry of Health, Brasilia ZC 70070-040, Brazil; giovanny.franca@saude.gov.br; 7 Physics Institute, Federal University of Bahia, Salvador, Bahia ZC 40210-340, Brazil; 8 Oswaldo Cruz Foundation, Gonçalo Moniz Institute, Salvador, Bahia ZC 40296-710, Brazil; qerenferreira@gmail.com; 9 Department of Communicable Diseases and Environmental Determinants of Health, Neglected, Tropical and Vector-Borne Diseases, Pan-American Health Organization/World Health Organization, Washington, WA 20037, USA; coelhogio@paho.org; 10 London School of Hygiene and Tropical Medicine, London WC1E 7HT, UK

**Keywords:** congenital Zika syndrome, microcephaly, case fatality rate, risks factors

## Abstract

Background: The clinical manifestations of microcephaly/congenital Zika syndrome (microcephaly/CZS) have harmful consequences on the child’s health, increasing vulnerability to childhood morbidity and mortality. This study analyzes the case fatality rate and child–maternal characteristics of cases and deaths related to microcephaly/CZS in Brazil, 2015–2017. Methods: Population-based study developed by linkage of three information systems. We estimate frequencies of cases, deaths, case fatality rate related to microcephaly/CZS according to child and maternal characteristics and causes of death. Multivariate logistic regression models were applied. Results: The microcephaly/CZS case fatality rate was 10% (95% CI 9.2–10.7). Death related to microcephaly/CZS was associated to moderate (OR = 2.15; 95% CI 1.63–2.83), and very low birth weight (OR = 3.77; 95% CI 2.20–6.46); late preterm births (OR = 1.65; 95% CI 1.21–2.23), Apgar < 7 at 1st (OR = 5.98; 95% CI 4.46–8.02) and 5th minutes (OR = 4.13; 95% CI 2.78–6.13), among others. Conclusions: A high microcephaly/CZS case fatality rate and important factors associated with deaths related to this syndrome were observed. These results can alert health teams to these problems and increase awareness about the factors that may be associated with worse outcomes.

## 1. Introduction

The emergence of the ZIKA virus (ZIKV) in Brazil, in the second half of 2014, was followed by the occurrence of an epidemic of microcephaly in newborns in 2015 [1], soon related to the congenital transmission of ZIKV by the Brazilian surveillance network [2]. Although ZIKV was identified in 1947 [3], its vertical transmission and consequent teratogenic effects had never been described before this epidemic [4]. This surprising and ominous outcome was initially detected after the discovery of a large concentration of cases in Recife, a city in the Northeast of Brazil, in October 2015 [1]. In the sequence, similar cases were identified in several other cities in this country and other countries in the Americas, where the ZIKV circulated [5]. Given this, The World Health Organization (WHO) declared a Public Health Emergency of International Concern, on 1 February 2016 [6].

Congenital microcephaly is characterized by the reduction greater than −2 standard deviations from the mean adjusted for gestational age, in the fetuses and newborn’s head circumference, which may be accompanied by abnormal brain development that causes complications such as delayed physical, neuromotor and cognitive development [7,8,9,10,11]. In addition, congenital ZIKV infection can cause several other damages, such as craniofacial disproportion, dysphagia, hyper-reflexia, spasticity, seizures, arthrogryposis, low birth weight and prematurity, among others [12,13,14,15,16]. Therefore, this spectrum makes it possible to recognize a new congenital syndrome that came to be named microcephaly/congenital Zika syndrome (microcephaly/CZS) [17,18,19].

Although microcephaly is the most evident alteration of CZS, other anomalies resulting from congenital ZIKV infection have been described, even in the absence of this clinical sign [20]. This wide spectrum of clinical manifestations of microcephaly/CZS and its harmful consequences on the child’s health and development, certainly increase vulnerability to childhood morbidity and mortality and may produce direct or indirect effects on their survival and development. Furthermore, the long-term effects on cognitive development remain partially unknown.

Considering that Brazil was the country most affected by microcephaly/CZS [21], and the availability of large population base datasets on vital and health events and, also, the scarcity of knowledge about the fatality from microcephaly/CZS, this study analyzes the case fatality rate and its associated factors in Brazil, from 2015 to 2017.

## 2. Materials and Methods

We carried out a population-based retrospective study by linking administrative data on microcephaly/CZS reports and live birth and death records in Brazil from 2015 to 2017.

### 2.1. Data Sources

Data were extracted from the Public Health Event Record (Registro de Eventos de Saúde Pública; RESP), Brazilian Live Births Information System (Sistema de Informações sobre Nascidos Vivos; SINASC) and the Mortality Information System (Sistema de Informações sobre Mortalidade; SIM). RESP is an online form developed by the Brazilian Ministry of Health with the purpose of recording data during public health emergencies. This instrument is used by all health services to register suspected cases of microcephaly and/or other Zika related congenital anomalies, including conditions related to infections during pregnancy, identified in prenatal care, childbirth and childcare [22]. From RESP we retained information on the final classification of the case, date of birth and the municipality of residence.

Live birth records are required by law and completed by the health worker who assisted the delivery. Records in SINASC cover 100% of the Brazilian territory [23], and include information on the mother, obstetric history, pregnancy characteristics and characteristics of the newborn. From SINASC we retained maternal characteristics such as age, marital status, race/color, education, number of fetuses, mode of delivery, fetal presentation, pregnancy duration and numbers of prenatal visits, and newborn characteristics such as age, sex, birth weight, Apgar score and congenital anomalies.

The SIM is based on the death declaration form, a legal document divided into blocks that has information on the dead person; place of death, characteristics of the mother (this block should be filled only in the case of fetal deaths or infant mortality) and cause of death using the International Classification of Diseases/ICD 10. It is important to note that in 2011, SIM coverage in Brazil was already 96.1% [24]. From SIM we retained age at death, date and cause of death.

### 2.2. Linkage Process

The linkage of these databases was performed using CIDACS-RL (Center for Data and Knowledge Integration for Health-Record Linkage), a novel record linkage tool developed to link big datasets at CIDACS [25]. This study is part of a broader project called “Zika and Congenital Zika Syndrome: Longitudinal Studies Platform”, which aims to enable long-term cohort studies and other epidemiological study designs to identify the spectrum of damage produced by microcephaly/CZS in the Brazilian population. This platform is anchored at CIDACS.

### 2.3. Procedures

We included all live births registered and classified in RESP as confirmed, probable and inconclusive cases of microcephaly/CZS and those that were still under investigation in December 2017. Cases under investigation were classified as inconclusive, as recommended by the Ministry of Health of Brazil (MoH) [8]. We now call “confirmed and possible” the set of cases of microcephaly/CZS included in our study. Considering the information recorded in the RESP, we excluded from this study fetal deaths, discarded cases and all cases that were positive to congenital TORCH (toxoplasmosis, other (HIV, syphilis, varicella zoster virus (VZV), etc.), rubella, cytomegalovirus (CMV) and herpes simplex virus-2 (HSV)).

Deaths of all children with confirmed and possible microcephaly/CZS registered in the RESP and linked with SIM, and of those not linked to this last information system, but which had the death record in the RESP, were considered. The death causes were grouped by categories of ICD 10.

### 2.4. Data Analysis

We estimated the percentage of cases, deaths and the case fatality rate of microcephaly/CZS by the year of occurrence for all five geographical regions of Brazil. The percentages of deaths according to basic and immediate causes were calculated.

Pearson’s Chi-square and Fisher’s exact tests, considering a 5% significance level (*p*-value < 0.05) were performed to test the differences between those microcephaly/CZS cases who evolved to deaths and those who did not die during the study period, according to clinical and sociodemographic characteristics at birth, pregnancy and delivery. Bivariate logistic regression was conducted to analyze the dependence between CZS deaths and variables considered for the study. Multivariate analysis was performed to estimate the odds ratio (OR) and their respective 95% confidence intervals, keeping in the final model only the independent variables, which have *p*-value < 0.05.

In all analyses, we considered only the cases and deaths that had valid data recorded.

### 2.5. Ethics Statement

This study analyzed unidentified data and was approved by the Federal University of Bahia Institute of Health Collective Research Ethics Committee (CAAE registration number no. 3,746,924).

## 3. Results

**Figure 1:**
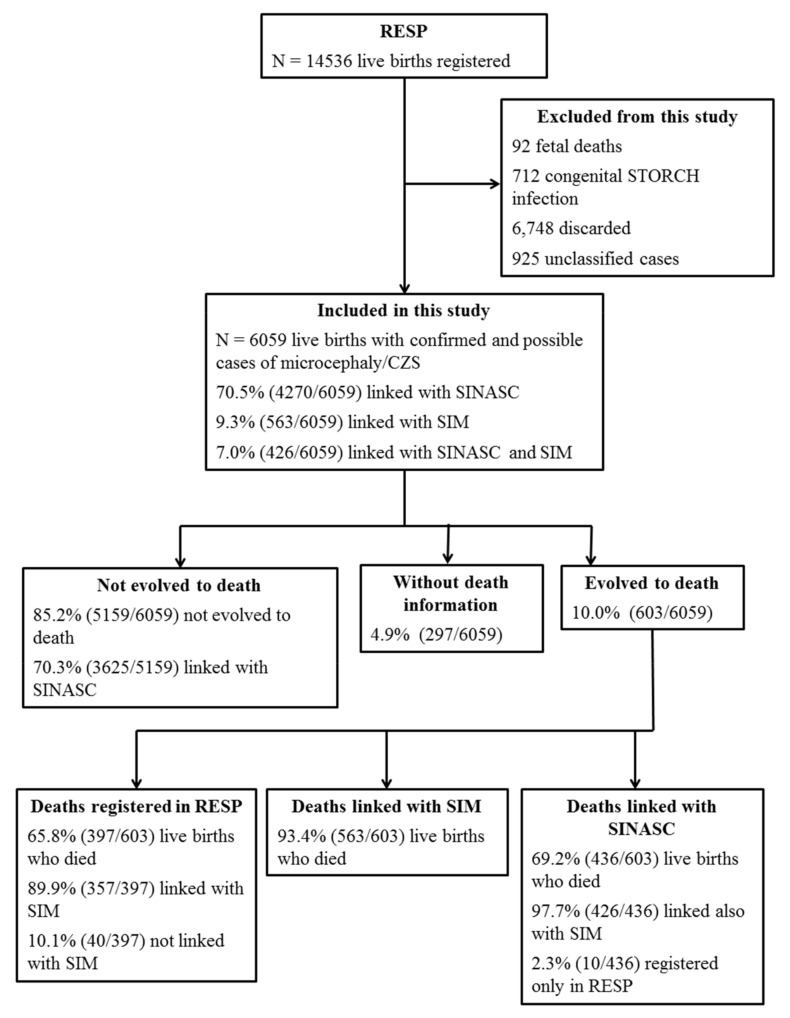
Flowchart of the selection process of notified cases and deaths by microcephaly/congenital Zika syndrome for the study of the case fatality rate related to this syndrome. Brazil, 2015 to 2017.

Of the 6059 live births with confirmed and possible cases of microcephaly/CZS, 603 of them evolved to death during the study period (Figure 1). The case fatality rate in this period was 10% (95% CI 9.2–10.7) overall, 2.9% in 2015, 10.3% in 2016 and 20.7% in 2017. The case fatality rate for only confirmed cases of CZS was 9.4% (95% CI 8.4–10.6), while for possible cases of this syndrome it was 10.4% (95% CI 9.4–11.5). From 557 deceased children that linked with SIM and had complete information on the age of death, aged between 0 and 2 years, 92.8% of deaths occurred under one-year-old, of which 52.6% in the neonatal period (early neonatal 38.2% and late neonatal 14.4%) and 40.2% in the post-neonatal period; 7.6% died at one year of age, and there was no record of death for children aged two years.

Of 563 liveborn children with confirmed and possible cases of microcephaly/CZS who were linked with a SIM record, 62.0% were classified into the Q00-Q99 (congenital malformations, deformities and chromosomal abnormalities) and 19.5% in the category P00-P96 (some disorders originating in the perinatal period) as the basic cause of death. In Table 1 we can observe that the highest proportions of immediate causes of deaths were: newborn’s respiratory failure—P28 (12.6%), unspecified septicemia—A41 (11.9%), bacterial septicemia—P36 (7.3 unspecified congenital malformations—Q89 (7.1%) and acute respiratory failure—J96 with 6.9%.

In 2015 and 2016, most liveborn children with confirmed and possible microcephaly/CZS cases were residents of the northeast region of the country (89.6% and 53.0%, respectively). In 2017, the southeast region registered the highest proportion of cases (43.9%). Considering the whole period studied, the highest proportion of deaths occurred in the northeast region ranging from 93.8% to 48.5% in the first and last year, respectively. However, the highest case fatality rate was observed in the north (13.6%) and centre-west (11.3%) regions (Table S1).

To compare the child–maternal characteristics of live births, we considered only those who had complete information for sex and age of death (only 7.6% did not have this information). When comparing characteristics of children with microcephaly/CZS, by survival status (non-survivors vs. survivors), we observed the following proportions: birth weight < 1500 g (16.9% vs. 3.0%), 1500–2499 g (48.5% vs. 31.3%), premature births/up to 31 weeks (10.0% vs. 2.4%), late preterm births/32–36 weeks (29.9% vs. 14.8%); Apgar < 7 in the 1st minute (58.4% vs. 10.0%) and 5th minute (30.6% vs. 2.2%), registered congenital anomaly (67.6% vs. 42.7%), and small for gestational age (49.8% vs. 34.9%) were higher in those who died than among those who survived during the study period, (*p* < 0.001; Table 2). We found that 59.5% of infants with microcephaly/CZS who died during the study period and who were born late premature and at term (34–36 and ≥37 weeks of gestation, respectively) had an Apgar score < 7 in the first minute. In its turn, only 7.9% of the survivors with that same gestational age had an Apgar score < 7 (data not shown in table).

The comparison of these two groups for maternal characteristics shows that there was no significant difference by maternal marital status, age group, race/color and educational level (Table 3).

In the final model (Table 4), the variables associated with the death of liveborn children with confirmed and possible microcephaly/CZS were moderate low birth weight/1500–2499 g (OR = 2.15; 95% CI 1.63–2.83), very low birth weight/500–1499 g (OR = 3.77; 95% CI 2.20–6.46), late preterm births/32–36 weeks (OR = 1.65; 95% CI 1.21–2.23), Apgar score < 7 at the 5th minute (OR = 4.13; 95% CI 2.78–6.13) and 1st minute (OR = 5.98; 95% CI 4.46–8.02), congenital anomaly (OR = 2.64; 95% CI 2.03–3.43), caesarean delivery (OR = 1.54; 95% CI 1.19–1.98) and twins or more babies (OR = 2.63; 95% CI 1.29–5.39).

## 4. Discussion

Our study was one of the few that comprehensively described case fatality rates among children with CZS, and was novel in describing the main and immediate cause of death and its associated factors. We observed that the case fatality rate among infants with microcephaly/CZS was 10%. The value of 20.7% observed in 2017 possibly reflects a distortion produced by the small number of cases that occurred in that year. About 40% of deaths occurred during the postneonatal period mainly coded as congenital malformations, deformities and chromosomal abnormalities or some disorders originating in the perinatal period. Although the northeast region recorded the highest number of deaths, the largest case fatality rates were observed in the north and Midwest regions. The main factor associated with deaths among children with microcephaly/CZS was the birth weight. Very low birth weight (VLBW) infants were 3.8 times more likely to die compared with those born with more than 2.5 kg. We observed that the Apgar score provided indication about the risk of death among infants with microcephaly/CZS; children with Apgar lower than 7 in the first minute were almost 6-fold more likely to die than those with Apgar equal or higher than 7.

Previous studies have estimated the microcephaly/CZS case fatality rate between 4% and 8%. However, they used the information of the death available only in RESP or another single data source [26,27]. Therefore, they would not be able to detect a child death that occurred after closing the case in RESP. In our study, we enhanced the RESP information by linking it to the Brazilian mortality information system. Thus, we were able to detect a more reliable number of deaths using information available in two independent data sources.

To our knowledge, there are no previous studies reporting characteristics associated with deaths among live births with microcephaly/CZS. However, VLBW has been spotted as a mortality risk factor among children with congenital abnormalities [28]. Even among live birth without congenital abnormalities, low birth weight, prematurity and small for gestational age are highly associated with morbidity and mortality [29].

We also observed that the odds ratio for death increased among those with Apgar lower than 7 in live births with microcephaly/CZS. The Apgar score indicates the condition of the infant moments after birth and may reflect a variety of underlying conditions and preceding events. Several components of the Apgar score, such as irritability and muscle tone, may be influenced by the CZS [30]. Consequently, the low Apgar score in this population may reflect the pathological syndrome presentation rather than impairment of the live birth as an otherwise healthy individual. Nevertheless, Apgar scores seem to provide useful prognostic information for the survival of children with microcephaly/CZS.

During the study period, Brazil had, on average 3.0 beds/1000 live births, a value higher than recommended by the Brazilian MoH [31] of 2 neonatal intensive care beds for 1000 live births. However, these resources are unevenly distributed across regions. Except for the south and southeast regions, all the other regions show a value lower than the parameter. In the northeast region, the most affected by the Zika epidemic, the average number of beds in that period was 1.6/1000 live births [32,33]. Therefore, it is plausible to hypothesize that this lack of neonatal intensive care beds to provide the necessary care for the most severe cases, right after birth, may have contributed to the high number of deaths, especially for the preterm low birth weight infants.

Similarly, those who were born late premature and at term, who represented almost 60% of children with microcephaly/CZS who died had an Apgar score < 7 in the first minute, a condition in which it is mandatory respiratory support at birth. Therefore, it is no accident that more than 50% of deaths in the first year of life occurred in the neonatal period.

Deaths related to microcephaly/CZS were not associated with age, race/color, marital status and maternal education. A possible explanatory hypothesis may be the similarity of the sociodemographic characteristics of the mothers who had children with this syndrome, who were mostly black or mixed, single and with low education. This predominance may be a consequence of greater protection in women (of childbearing age and pregnant women) from more affluent social classes against the bites of *Aedes aegypti*. Besides, we cannot rule out the possibility of greater access by these women to a safe abortion procedure, even though this practice is considered illegal in Brazil.

The main novelty of our study is the use of nationwide data with a large sample size. In addition, the information available in RESP was enhanced by the other two sources of data (SIM and SINASC), which provided information to assess the basic and immediate causes of deaths and the birth information. There are, however, limitations particularly, because at the beginning of the epidemic, the health services network did not have specific diagnostic tests for ZIKV in newborns. First, as the study included inconclusive cases, misclassification is possible. At birth, a criterion for the suspicion of microcephaly/CZS is the head circumference, even in the absence of the newborn’s craniofacial disproportion. Therefore, small children for gestational age without microcephaly could be included in the case definition. Second, there may also have been underreporting in the RESP of children diagnosed with microcephaly/CZS, and among children without detectable malformations, that may only manifest CZS sequels with a delay in neuromotor development or seizures. Third, it is possible that mild cases of microcephaly/CZS were not registered in the RESP and therefore are not part of our study. Linkage error could have occurred due to a lack or unreliable essential information, which could underestimate our measure of case fatality rate. Thus, information bias (classification and registration biases) may have occurred. We still have another limitation, which was the fact that they did not assess the severity of microcephaly as a risk factor for death for these children.

Despite these limitations, our study presented valuable information on deaths among microcephaly/CZS live births. The presented results will be usefully used to alert and direct health teams to the causes of these deaths and generate awareness about the factors that may be associated with worse outcomes. We recommend further research to explore the effects of microcephaly/CZS on morbidity aimed to seek clinical support alternatives that can bring benefits to children with this syndrome. Finally, given that countless urban centers in Brazil and many other countries in the Americas continue to be infested by mosquitoes of the *Aedes* genus, especially *Aedes aegypti,* the risk of ZIKV circulation, in endemic and/or epidemic form, remains, imposing the need for permanent reinforcement of individual protection measures, especially for pregnant women and women of childbearing age, and the strengthening and improvement of vector control actions aimed at preventing new cases of microcephaly/CZS.

## Figures and Tables

**Table 1:** Number and percentage of deaths of children with microcephaly/congenital Zika syndrome (confirmed and possible ^a^), according to the immediate cause. Brazil, 2015–2017.

Immediate (or Terminal) Cause of Death (ICD 10)	*N*	%
Respiratory insufficiency of the newborn (P28)	71	12.6
Unspecified septicemia (A41)	67	11.9
Acute Resp. insufficiency; Unspecified Resp. insuffic. (J96)	39	6.9
Bacterial septicemia, unspecified newborn (P36)	41	7.3
Unspecified Cong malformations (Q89)	40	7.1
Conditions originating in the specific period (P96)	26	4.6
Microcephaly (Q02)	19	3.4
Cardiog. shock; Shock Hipov; Shock not specified (R57)	21	3.7
Other specific symptoms and general signs (R68)	17	3.0
Asphyxia at birth, unspecified (P21)	18	3.2
Insufficiency Neonatal heart (P29)	12	2.1
Dextrocardia; Malform. does not specify heart (Q24)	10	1.8
Bronchopneumonia. (not specified (J18))	10	1.8
Pulmonary hemorrhages originating in the perinatal period (P26)	10	1.8
Malform Cong non-brain (Q04)	8	1.4
Respiratory Arrest (R09)	8	1.4
Other causes poorly defined and unspecified (R99)	8	1.4
Unspecified cardiac arrest (I46)	6	1.1
Very low birth weight; preterm newborn (P07)	7	1.2
Other Causes	125	22.2
**TOTAL**	563	100.0

Source: SIM (mortality information system). ^a^ probable and inconclusive cases and those under epidemiological investigation 60 days after the start of this activity.

**Table 2:** Number and percentage of live birth with microcephaly/congenital Zika syndrome (confirmed and possible ^a^) who died and who did not die according to the child’s clinical characteristics and sex. Brazil, 2015–2017.

Live Birth with Microcephaly/CZS	Nonsurvivors						Survivors					
Sex	Male		Female		Total ^b^		Male		Female		Total ^b^	
Characteristics	*N*	%	*N*	%	*N*	%	*N*	%	*N*	%	*N*	%
Birth weight (grams)												
500–1499	35	18.4	38	16.4	73	16.9	44	2.8	63	3.1	107	3.0
1.500–2499	86	45.3	115	49.6	209	48.5	429	26.9	705	34.8	1134	31.3
≥2500	69	36.3	79	34.0	149	34.6	1123	70.4	1257	62.1	2381	65.7
Prematurity												
<32	19	10.4	22	10.2	41	10.0	39	2.5	46	2.3	85	2.4
32–36	62	33.9	59	27.3	122	29.9	245	15.8	277	14.1	522	14.8
≥37	102	55.7	135	62.5	245	60.1	1264	81.7	1647	83.6	2912	82.8
Apgar at 1 min												
<7	111	60.0	125	55.6	244	58.4	152	9.9	199	10.2	351	10.0
7–10	74	40.0	100	44.4	174	41.6	1388	90.1	1760	89.8	3149	90.0
Apgar at 5 min												
<7	64	34.8	59	26.1	128	30.6	35	2.3	41	2.1	76	2.2
7–10	120	65.2	167	73.9	290	69.4	1507	97.7	1918	97.9	3426	97.8
Congenital Anomalies												
Yes	130	68.8	151	65.4	290	67.6	680	43.4	840	42.2	1521	42.7
No	59	31.2	80	34.6	139	32.4	888	56.6	1151	57.8	2039	57.3
Small for gestational age												
No	47	40.9	79	55.6	133	50.2	278	59.7	511	68.5	789	65.1
Yes	68	59.1	63	44.4	132	49.8	188	40.3	235	31.5	423	34.9

Source: SINASC (live birth information system); SIM (mortality information system). All variables showed statistically significant difference between children with microcephaly/CZS who died and those who did not die. *p* < 0.001, except sex *p* = 0.724. ^a^ probable and inconclusive cases and those under epidemiological investigation 60 days after the start of this activity. ^b^ Including missing data.

**Table 3:** Number and percentage of live birth with microcephaly/congenital Zika syndrome (confirmed and possible ^a^) who died and who did not die according to maternal sociodemographic and health care characteristics. Brazil. 2015–2017.

Live Birth with Microcephaly/CZS	Nonsurvivors		Survivors		*p*
Maternal Characteristics	*N*	%	*N*	%	
Age group (years)					
15–19	101	23.5	786	22.0	
20–29	206	48.0	1783	49.8	NS ^b^
30–50	122	28.5	1009	28.2	
Marital status					
Single/widow/divorced	245	57.0	1891	52.8	
Married/union	185	43.0	1690	47.2	NS ^b^
Race/color					
White	68	17.1	589	17.1	NS ^b^
Black/Mixed	318	80.1	2824	82.2	
Yellow/indigenous ^c^	11	2.8	25	0.7	
Educational Level					
Illiterate	3	0.7	22	0.6	
Elementary I	30	7.1	240	6.7	
Elementary II	352	83.2	2928	82.1	
Incomplete High school	16	3.8	139	3.9	
Complete High School	22	5.2	239	6.7	NS ^b^
Type of delivery					
Vaginal	177	40.8	1977	54.6	<0.001
Caesarean section	257	59.2	1644	45.4	
Pregnancy type					
Only	420	96.8	3575	98.7	0.005 *
Twins or more	14	3.2	46	1.3	
Pregnancy duration (weeks)					
<34	74	18.1	180	5.1	
34–36	89	21.8	427	12.1	
≥37	245	60.1	2912	82.8	<0.001
Numbers of prenatal visits					
<7	227	53.0	1508	42.0	
≥7	201	47.0	2085	58.0	<0.001
Newborn presentation					
Cephalic	350	82.9	3350	94.4	<0.001 *
Pelvic	70	16.6	191	5.4	
Transversal	2	0.5	6	0.2	

Source: SINASC (live birth information system), SIM (mortality information system). Refer only to cases with information registered. ^a^ probable and inconclusive cases and those under epidemiological investigation 60 days after the start of this activity. ^b^ NS: Not significant. ^c^ Yellow and indigenous categories were excluded from the analysis. * Fisher’s exact test.

**Table 4:** Odds ratios (ORs) and confidence intervals (95% CIs) obtained by logistic regression for the association between deaths of live birth with congenital Zika syndrome (confirmed and possible ^a^) and potential risk factors. Brazil, 2015–2017.

Model Variables	Crude		Adjusted	
	OR	95% CI	OR	95% CI
Birth weight (g)				
2500–7000	1.00	...	1.00	...
1500–2499	2.06	1.56–2.71	2.15	1.63–2.83
500–1499	3.56	2.07–6.11	3.77	2.20–6.46
Number of week of gestation				
≥37	1.00	...	1.00	...
32–36	1.65	1.21–2.25	1.65	1.21–2.23
<32	1.15	0.59–2.23	1.27	0.66–2.42
Apgar at 5 min				
7–10	1.00	...	1.00	...
<7	4.24	2.84–6.32	4.13	2.78–6.13
Apgar at 1 min				
7–10	1.00	...	1.00	...
<7	5.91	4.40–7.95	5.98	4.46–8.02
Congenital anomaly				
No	1.00	...	1.00	...
Yes	2.69	2.06–3.50	2.64	2.03–3.43
Delivery type				
Vaginal	1.00	...	1.00	...
Caesarean	1.59	1.23–2.06	1.54	1.19–1.98
Number of babies				
Singleton	1.00	...	...	...
Twins or more	2.75	1.34–5.64	2.63	1.29–5.39
Numbers of prenatal visits				
≥7	1.00	...	...	...
<7	1.26	0.97–1.63	...	...

Source: SINASC (live birth information system); SIM (mortality information system); RESP (public health events register). ^a^ Probable and inconclusive cases and those under epidemiological investigation 60 days after the start of this activity. Refer only to cases with information registered.

## Supplementary Materials

The following are available online at https://www.mdpi.com/1999-4915/12/11/1228/s1, Table S1: Number and percentage of cases and deaths and case fatality rate of live birth with Congenital Zika Syndrome (confirmed and possible ^a^) according year of occurrence and regions. Brazil, from 2015 to 2017.

## References

[1] Teixeira M.G., da Conceição NCosta M., de Oliveira W.K., Nunes M.L., Rodrigues L.C. (2016). The Epidemic of Zika Virus–Related Microcephaly in Brazil: Detection, Control, Etiology, and Future Scenarios. Am. J. Public Health.

[2] Rasmussen S.A., Jamieson D.J., Honein M.A., Petersen L.R. (2016). Zika Virus and Birth Defects—Reviewing the Evidence for Causality. N. Engl. J. Med..

[3] Dick G.W.A., Kitchen S.F., Haddow A.J. (1952). Zika Virus (I). Isolations and serological specificity. Trans. R. Soc. Trop. Med. Hyg..

[4] Krauer F., Riesen M., Reveiz L., Oladapo O.T., Martínez-Vega R., Porgo T.V., Haefliger A., Broutet N.J., Low N., WHO Zika Causality Working Group (2017). Zika Virus Infection as a Cause of Congenital Brain Abnormalities and Guillain–Barré Syndrome: Systematic Review. PLoS Med..

[5] Pan American Health Organization (2016). Timeline of Emergence of Zika Virus in the Americas.

[6] World Health Organization WHO WHO Statement on the First Meeting of the International Health Regulations. Emergency Committee on Zika Virus and Observed Increase in Neurological Disorders and Neonatal Malformations. www.who.int/mediacentre/news/statements/2016/1st-emergency-committee-zika/en/.

[7] Alvarado-Socarras J.L., Idrovo Á.J., Contreras-García G.A., Rodriguez-Morales A.J., Audcent T.A., Mogollon-Mendoza A.C., Paniz-Mondolfi A. (2018). Congenital microcephaly: A diagnostic challenge during Zika epidemics. Travel Med. Infect. Dis..

[8] Ministério da Saúde do Brasil, Secretaria de Vigilância em Saúde, Secretaria de Atenção à Saúde (2017). Orientações Integradas de Vigilância e Atenção à Saúde no Âmbito da Emergência de Saúde Pública de Importância Nacional: Procedimentos Para o Monitoramento das Alterações no Crescimento e Desenvolvimento a Partir da Gestação Até a Primeira Infância, Relac.

[9] Ximenes A.S.F.C., Pires P., Werner H., Jungmann P.M., Rolim Filho E.L., Andrade E.P., Lemos R.S., Peixoto A.B., Zare Mehrjardi M., Tonni G. (2019). Neuroimaging findings using transfontanellar ultrasound in newborns with microcephaly: A possible association with congenital Zika virus infection. J. Matern. Neonatal Med..

[10] Araujo E., Carvalho F.H.C., Tonni G., Werner H. (2017). Prenatal imaging findings in fetal Zika virus infection. Curr. Opin. Obstet. Gynecol..

[11] Carvalho F.H.C., Cordeiro K.M., Peixoto A.B., Tonni G., Moron A.F., Feitosa F.E.L., Feitosa H.N., Araujo Júnior E. (2016). Associated ultrasonographic findings in fetuses with microcephaly because of suspected Zika virus (ZIKV) infection during pregnancy. Prenat. Diagn..

[12] Bertolli J., Attell J.E., Rose C., Moore C.A., Melo F., Staples J.E., Kotzky K., Krishna N., Satterfield-Nash A., Pereira I.O. (2020). Functional outcomes among a cohort of children in northeastern Brazil meeting criteria for follow-up of congenital Zika virus infection. Am. J. Trop. Med. Hyg..

[13] Oliveira-Filho J., Felzemburgh R., Costa F., Nery N., Mattos A., Henriques D.F., Ko A.I., Fukuda J.S., Khouri R., Pereira L.P. (2018). Seizures as a complication of congenital Zika syndrome in early infancy. Am. J. Trop. Med. Hyg..

[14] Leal M.C., van der Linden V., Bezerra T.P., de Valois L., Borges A.C.G., Antunes M.M.C., Brandt K.G., Moura C.X., Rodrigues L.C., Ximenes C.R. (2017). Characteristics of dysphagia in infants with microcephaly caused by congenital zika virus infection, Brazil, 2015. Emerg. Infect. Dis..

[15] Carvalho-Sauer R., Costa M.D.C.N., Barreto F.R., Teixeira M.G. (2019). Congenital Zika Syndrome: Prevalence of low birth weight and associated factors. Bahia, 2015–2017. Int. J. Infect. Dis..

[16] Del Campo M., Feitosa I.M.L.L., Ribeiro E.M., Horovitz D.D.G.G., Pessoa A.L.S.S., França G.V.A.A., García-Alix A., Doriqui M.J.R.R., Wanderley H.Y.C.C., Sanseverino M.V.T.T. (2017). The phenotypic spectrum of congenital Zika syndrome. Am. J. Med. Genet..

[17] Moore C.A., Staples J.E., Dobyns W.B., Pessoa A., Ventura C.V., da Fonseca E.B., Ribeiro E.M., Ventura L.O., Neto N.N., Arena J.F. (2017). Characterising the pattern of anomalies in congenital zika syndrome for pediatric clinicians. JAMA Pediatr..

[18] Chan J.F.W.W., Choi G.K.Y.Y., Yip C.C.Y.Y., Cheng V.C.C.C., Yuen K.Y. (2016). Zika fever and congenital Zika syndrome: An unexpected emerging arboviral disease. J. Infect..

[19] Costello A., Dua T., Duran P., Gülmezoglu M., Oladapo O.T., Perea W., Pires J., Ramon-Pardo P., Rollins N., Saxena S. (2016). Defining the syndrome associated with congenital Zika virus infection. Bull. World Health Organ..

[20] Adachi K., Romero T., Nielsen-Saines K., Pone S., Aibe M., Barroso De Aguiar E., Sim M., Brasil P., Zin A., Tsui I. (2020). Clinical Infectious Diseases Early Clinical Infancy Outcomes for Microcephaly and/or Small for Gestational Age Zika-Exposed Infants. Clin. Infect. Dis..

[21] Barbeito-Andrés J., Schuler-Faccini L., Garcez P.P. (2018). Why is congenital Zika syndrome asymmetrically distributed among human populations?. PLoS Biol..

[22] Ministério da Saúde do Brasil RESP-Registro de Eventos Em Saúde Pública. http://www.resp.saude.gov.br/microcefalia#/painel.

[23] De Bonilha E.A., Vico E.S.R., de Freitas M., Barbuscia D.M., Galleguillos T.G.B., Okamura M.N., Dos Santos P.C., de Lira M.M.T.A., Torloni M.R. (2018). Cobertura, completude e confiabilidade das informações do Sistema de Informações sobre Nascidos Vivos de maternidades da rede pública no município de São Paulo, 2011. Epidemiol. E Serv. Saude Rev. Do Sist. Unico Saude Do Bras..

[24] Ministério da Saúde do Brasil, Secretaria de Vigilância em Saúde (2013). Coordenação Geral de Informações e Análise Epidemiológica. Sistema de Informações Sobre Mortalidade-SIM Consolidação da Base de Dados de 2011.

[25] Almeida D., Gorender D., Ichihara M.Y., Sena S., Menezes L., Barbosa G.C.G., Fiaccone R.L., Paixão E.S., Pita R., Barreto M.L. (2020). Examining the quality of record linkage process using nationwide Brazilian administrative databases to build a large birth cohort. BMC Med. Inform. Decis. Mak..

[26] Mendes Neto N.N., da Silva Maia J.T., Zacarkim M.R., Queiroz I., Labeaud A.D., Aronoff D.M. (2018). Perinatal Case Fatality Rate Related to Congenital Zika Syndrome in Brazil: A Cross-Sectional Study. Pediatr. Neurol..

[27] Da Cunha A.J.L.A., de Magalhães-Barbosa M.C., Lima-Setta F., Medronho R.d.A., Prata-Barbosa A. (2017). Microcephaly Case Fatality Rate Associated with Zika Virus Infection in Brazil: Current Estimates. Pediatr. Infect. Dis. J..

[28] Kawasaki H., Yamada T., Takahashi Y., Nakayama T., Wada T., Kosugi S. (2020). Epidemiology of Birth Defects in Very Low Birth Weight Infants in Japan. J. Pediatr..

[29] Katz J., Lee A.C.C., Kozuki N., Lawn J.E., Cousens S., Blencowe H., Ezzati M., Bhutta Z.A., Marchant T., Willey B.A. (2013). Mortality risk in preterm and small-for-gestational-age infants in low-income and middle-income countries: A pooled country analysis. Lancet.

[30] Pessoa A., Linden V.V.D., Yeargin-allsopp M., Costa D., Van Der Linden V., Yeargin-allsopp M., Carvalho M.D.C.G., Ribeiro E.M., Van Naarden Braun K., Durkin M.S. (2018). Motor Abnormalities and Epilepsy in Infants and Children With Evidence of Congenital Zika Virus Infection. Pediatrics.

[31] Ministério da Saúde do Brasil. Gabinete do Ministro Portaria N° 930, de 10 de Maio de 2012. http://bvsms.saude.gov.br/bvs/saudelegis/gm/2012/prt0930_10_05_2012.html.

[32] Ministério da Saúde do Brasil DATASUS-Informações de Saúde (TABNET) Sobre Rede Assistencial. http://www2.datasus.gov.br/DATASUS/index.php?area=0204&id=1479586&VObj=http://tabnet.datasus.gov.br/cgi/deftohtm.exe?cnes/cnv/leiuti.

[33] Ministério da Saúde do Brasil DATASUS TabNet Win32 3.0: Nascidos Vivos. http://tabnet.datasus.gov.br/cgi/deftohtm.exe?sinasc/cnv/nvuf.def.

