# Supplementary material for: Case Fatality Rate Related to Microcephaly Congenital Zika Syndrome and Associated Factors: A Nationwide Retrospective Study in Brazil
Source: Viruses. 2020 Oct 29;12(11):1228. doi: 10.3390/v12111228 (PMC7692842; doi:10.3390/v12111228)
Supplement: Supplementary file 1 [file viruses-12-01228-s001.pdf]

viruses

MDPI

Table S1. Number and percentage of cases and deaths and case fatality rate of live birth with Congenital Zika Syndrome (confirmed and possible[a]) according year of occurrence and regions. Brazil, from 2015 to 2017.

**Year**	**2015**					2016					2017					TOTAL				
	**Case**		Death		Fat.	Case		Death		Fat.	Case		Death		Fat.	Case		Death [b]		Fat.
**Regions**	**N**	**%**	N	%	%	N	%	N	%	%	N	%	N	%	%	N	%	N	%	%
North	42	1, 1.9	2	3.1	4.8	282	9.7	29	9.6	10.3	72	7.5	20	10.1	27.8	396	6.5	54	9.0	13.6
Northeast	1956	89.6	60	93.8	3.1	1547	53.0	185	61.5	12.0	315	32.9	96	48.5	30.5	3818	63.0	366	60.7	9.6
Center West	49	2.2	-	-	-	264	9.0	22	7.3	8.3	122	12.8	23	11.6	18.9	435	7.2	49	8.1	11.3
Southeast	134	6.1	2	3.1	1.5	814	27.9	65	21.6	8.0	420	43.9	56	28.3	13.3	1368	22.6	130	21.6	9.5
South	2	0.1	-	-	-	12	0.4	-	-	-	28	2.9	3	1.5	10.7	42	0.7	4	0.7	9.5
Brazil	2183	36.0	64	11.4	2.9	2919	48.2	301	53.5	10.3	957	15.8	198	35.2	20.7	6059	100.0	603	100.0	10.0

Source: RESP (Public Health Events Register); SIM (Mortality Information System)

a-probable and inconclusive cases and those under epidemiological investigation 60 days after the start of this activity.

b - Includes 40 records not linked to SIM
